# A deep learning approach to fight illicit trafficking of antiquities using artefact instance classification

**DOI:** 10.1038/s41598-022-15965-2

**Published:** 2022-08-05

**Authors:** Thomas Winterbottom, Anna Leone, Noura Al Moubayed

**Affiliations:** 1 Department of Computer Science, Durham University, Durham, UK; 2 Department of Archaeology, Durham University, Durham , UK

**Keywords:** Archaeology, Computational science

## Abstract

We approach the task of detecting the illicit movement of cultural heritage from a machine learning perspective by presenting a framework for detecting a *known artefact* in a new and unseen image. To this end, we explore the machine learning problem of *instance classification* for large archaeological images datasets, i.e. where each individual object (instance) is itself a class that all of the multiple images of that object belongs. We focus on a wide variety of objects in the Durham Oriental Museum with which we build a dataset with over 24,502 images of 4332 unique object instances. We experiment with state-of-the-art convolutional neural network models, the smaller variations of which are suitable for deployment on mobile applications. We find the *exact object instance* of a given image can be predicted from among 4332 others with ~ 72% accuracy, showing how effectively machine learning can detect a known object from a new image. We demonstrate that accuracy significantly improves as the number of images-per-object instance increases (up to ~ 83%), with an ensemble of classifiers scoring as high as 84%. We find that the correct instance is found in the top 3, 5, or 10 predictions of our best models ~ 91%, ~ 93%, or ~ 95% of the time respectively. Our findings contribute to the emerging overlap of machine learning and cultural heritage, and highlights the potential available to future applications and research.

## Introduction

Punctual identification of objects is an essential aspect of the fight against illicit trafficking, as police can only intervene and seize an object if they can prove it is looted by identifying its original provenance. As such, substantial efforts have been undertaken to both draw attention to and curtail the loss of cultural heritage through looting and trafficking over the past 50 years. The “Convention of the Means of Prohibiting and Preventing the Illicit Import, Export and Transfer of Ownership of Cultural Property” by UNESCO in 1970^1^ outlines practices that have been favoured by recent investments into antiquities, where the purchased artefacts are expected to increase in value. The practices outlined in this convention have been progressively adapted and developed over time in response to large-scale events of concern. Notable attacks on museums and widespread looting following the Soviet withdrawal from Afghanistan^2^ motivated the “Convention of Stolen and Illegally exported Cultural Objects” in 1995^3^ to specifically reinforce the 1970 convention. Despite these efforts, illicit trafficking has further developed in scale and strategy following the wars in Iraq, Syria, and Libya, and after the Arab spring in North Africa. It has been estimated that the overall income of the illicit trafficking is around $2.2 Billion USD every year^4^. Though it is difficult fully understand the details of such a black market, it is clear that it represents a major source of income for international terrorism. Illicit trafficking is organised through networks, and despite the differences between countries the process is always fundamentally the same: Goods are stolen and transferred with the provenance changed a few times so that the origin of the artefact cannot be determined^4^. In this process the one of the keys to the trafficker’s success are photographs. The object is photographed several times in different orientations and scenarios such that the object becomes extremely challenging to identify without expert intervention. However, the intractably difficult task of manually detecting an ever increasing set of at-risk artefacts at every potential checkpoint necessitates an automated solution. The creation of a system which identifies an *object* from an unseen image (despite variations in lighting or orientation) is seen as essential. Such a tool would be extremely important for customs police by enabling new photos of potential illicitly-trafficked artefacts to be rapidly compared to a very large database of at-risk objects *simultaneously*, while not being vulnerable to deliberate variations in the submitted photographs. Furthermore, such a system could be used to search and detect stolen objects in large online auction houses. By formulating this scenario as the problem of ‘detecting the *exact* object a never-before-seen image depicts by leveraging existing *known* images of that object’—that is, to detect the *exact object instance* of an image—we find a task that machine learning and computer vision are well equipped to solve. Using state-of-the-art deep convolutional neural networks (CNNs), we solve the task of *object instance* classification on a large dataset of images of diverse cultural heritage artefacts from the Durham Oriental Museum. The dataset consists of 24,502 images of 4332 different objects from ancient Greece, ancient Rome, ancient Egypt, and a collection of post-medieval Oriental artefacts. To solve this 4332-class classification task, we construct a neural network model by finetuning on pretrained EfficientNet^5^ and ResNet Rescaled^6^ models with further ablation on Inception-v3^7^/v4^8^. Our experiments find that the *exact* correct instance can be identified with 72.12% accuracy—from amongst 4332 (i.e. a baseline accuracy of 0.023%)—, and that increasing the minimum number of images-per-instance from 3 to 6 further increases accuracy up to 83.28%. Though our highest accuracy comes from a collaborative ensemble of all models (84.02%), the smaller variations of the EfficientNet model maintain an accuracy of $$\sim$$ 81% while being suitable for inference on mobile devices. We find that in the case of incorrect classifications, the correct answer is often still found in the top few guesses, i.e. we find the top-3, top-5, and top-10 accuracies of our strongest models are 90.53%, 92.82%, and 94.80% respectively. This allows our framework the flexibility to purchase an even higher accuracy with a small number of additional objects for manual review. Our analysis includes both: (1) a breakdown of model performance by the subcategories of the dataset, where we find that objects from the ‘Oriental’ and ‘Egyptian’ subsections are most accurately classified; (2) a breakdown of model performance by the number of images each instance has, where we find that although instances with higher image counts are significantly easier to classify, the accuracy improvements begin to diminish at 8 images-per-instance. Our findings suggest that though more images-per-instance will allow more accurate detection, 8 images may be an ideal ‘sweet spot’ to aim for if resources are limited. Though the very largest EfficientNet models perform best, we find that smaller models—suitable for inference of mobile devices—are within 2% accuracy of the larger models for a fraction of the computational cost.

“Background” section briefly introduces convolutional neural networks (CNNs) and their use in image-based machine learning tasks (computer vision), and highlight related machine learning vision tasks in cultural heritage. “Methods” section describes both the dataset and convolutional neural network (CNN) models we use in our experiments. “Results” section contains the experimental results of our instance classification task. “Discussion” section discusses our results and how they represent a proof-of-concept for a known artefact detection framework. “Limitations” section highlights the limitations of our approach and outlines desirable properties for future datasets.

## Background

### Convolutional neural networks

**Figure 1:**
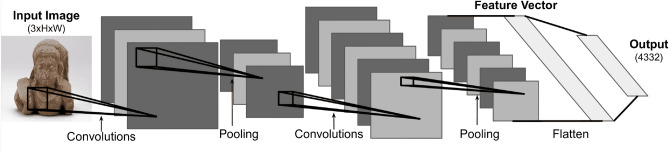
CNN architecture with an RGB image input and desired outputs representing votes for objects in of our instance classification task.

CNNs refer to a neural network substantially comprised of ‘convolution’ layers that excel at processing grid-like information i.e. images. Convolution layers scan a weighted kernel across the regions of its input, and for each region outputs dot product of the weighted kernel and the given input region (Fig. 1). Convolution layers often contain multiple learnable kernels, allowing each kernel to implicitly learn to detect different local patterns. CNN-based modelling approaches have remained state-of-the-art for processing and classifying images in machine learning over the decade. Like any other computer-based neural network architecture, CNNs are comprised of a sequential layers of artificial ‘neurons’ that take numerical values as inputs, and then output another numerical value to the next layer which is controlled by ‘learnable’ weights and biases (i.e. weights are continually adjusted automatically by the training algorithm). The numerical inputs are propagated through the entire network, resulting in a final numerical output that must be interpreted in some way as an ‘answer’ to the input, with the weights adjusted such that correct ‘answer’ output for the specific given input is encouraged. The key concept to neural networks is that all inputs and outputs are purely numerical. A neural network can be applied to a task *if the inputs and outputs can be* *effectively and sufficiently* *represented numerically*. For our task of instance classification, we ‘classify’ a HxW dimensional image as a depiction of one of 4332 unique objects. The input image can be represented *numerically* as an RGB image object (3 colour channels) of pixel values (each 0–255) i.e. an array of dimensions (3, *H*, *W*) of numbers. Note that images are convenient to *effectively* represent numerically as they are wholly described by the pixel values that comprise them, whereas representing a word or sentence numerically is less intuitively obvious^9^. We now design the *output* of the neural network such that our task can be *effectively* represented numerically. As our task of instance classification here is an attempt to distinguish between 4332 different objects, we can *choose* to have our final layer give 4332 different outputs, and then *choose to interpret* each of those individual outputs as a vote for one of the objects that the input image might be depicting. During training, a ‘loss’ (a numerical measure of how wrong the network is) is calculated from the outputs for each example in the training set (in ‘batches’) by comparing it to the correct answer. Each weight in the network is then updated to compensate for this loss. The training process is repeated over the entire training set for a number of ‘epochs’, until its ability to generalise to the task is tested on an unseen ‘test’ dataset. The architectural designs of CNNs have evolved over the past 20 years through increasing model size (i.e. number of layers or ‘depth’) and architectural design innovations. The most notable of which in chronological order are: LeNet^10^, a very early, small, and simplistic design; AlexNet^11^ a much deeper design with more channels and a variety of kernel sizes; InceptionNet^12^, deeper still with extra classification layers partway through the network to stop the changes in weights (gradients) from vanishing; VGG^13^ which replaces larger convolutional kernels with smaller ones stacked on top of eachother to more efficiently cover the same receptive field; Inception-v3^7^ decomposes *nxn* kernels into 1*xn* and *nx*1; ResNet^14^ and Inception-v4^8^ which introduced ‘skip connections’ allowing information to ‘skip’ forward to avoid vanishing gradients given the increasing depth, and most recently EfficientNet^5^, which focused on a more efficient architecture using a variety of small innovations (e.g. a new ‘activation function’) yielding increased performance with a comparatively smaller size.

### Machine learning in cultural heritage

Recent research efforts have surveyed applications of machine learning for specific domains of cultural heritage such as historical document processing^15^ and analysis of dance techniques^16^. Our work is similar in motivation to efforts monitoring sales activity of antiquities on auctions sites (*e.g.* eBay) through machine learning approaches such as named entity recognition^17^ and natural language processing^18^. In contrast to such text-based applications, our work instead focuses on *images* of specific objects and artefacts, and thus a CNN-based approach. Jain et al.^19^ use the ResNet-50^14^ CNN architecture to classify Indian classical dance forms. Azhar et al.^20^ use a collaborative ‘ensemble’ of multiple CNNs to improve their classification algorithm for batik images. See Fiorucci et al.^21^ for a broad review of language/vision-based machine learning approaches for multiple domains of cultural heritage. These research efforts focus on classification of images, and counteracting illicit trade of antiquities using textual data. To the best of our knowledge, ours is the first work to leverage instance classification of images on data focusing on cultural heritage artefacts and their protection. See Table 1 for a comparative breakdown of our methodology compared to that of related research.

**Table 1:** A comparative categorical breakdown of our approach compared to similar research.

	Cultural heritage	Data modality	Task	Vs. illicit trafficking
Ours	✓	Vision	Instance classification	✓
^15^	✓	Vision + Language	N/A (srvey)	✗
^16^	✓	Vision	N/A (survey)	✗
^17^	✓	Language	Named entity recognition	✓
^18^	✓	Language	Language analysis	✓
^19^	✓	Vision	Dance form classification	✗
^20^	✓	Vision	Batik classification	✗
^21^	✓	Vision + Language + more	N/A (survey)	✓ and ✗

## Methods

### Dataset

**Figure 2:**
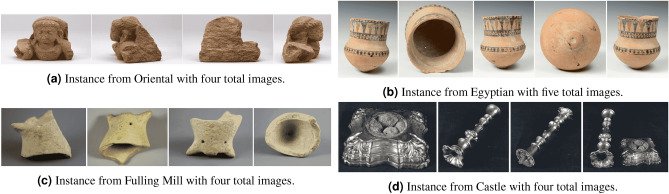
Images from instances of each of the four subcategories: ‘Oriental’, ‘Egyptian’, ‘Fulling Mill’, and ‘Castle’.

Using images from the digital collection of the Durham Oriental Museum, we create a dataset of individual objects (instances) with multiple images each. We note that the instance classification requires a model correctly classify a *new and unseen image* of an object it has *already seen*. With this in mind, we require that each instance has at least 3 different images to ensure that the object is adequately represented in both the training, and validation or test splits. After discarding instances with 2 or less images, the dataset has 24,502 images of 4332 object instances, i.e. it is 4332-class classification task. Over 75% of the *instances* in our dataset have between three and six images (see Table 2a). Note that this range equates to only 59% of total *images*. We focus primarily on the distribution of image counts of individual instances in service of our instance classification task and it’s implication for cultural heritage. However, the images used in this dataset are also *categorically* divided (by the collections they are originate from) into four subsections: Post medieval ‘Oriental’ (Fig. 2a), ancient ‘Egyptian’ (Fig. 2b), ‘Fulling Mill’ i.e. ancient Greece and ancient Rome (Fig. 2c), and ‘Castle’ (Fig. 2d; see Table 2b). Images of individual object instance are from different orientations and views, leading certain object shapes to cause challenging scenarios as seen in the 4th image of Fig. 2c. The images of other instances feature individual parts of a whole object as in Fig. 2d, necessitating that models learn subtle stylistic patterns at different resolutions in addition to shapes and dimension.

**Table 2:** Structural breakdown of the dataset used in our experiments.

Images per instance	Instances	Images
**(a) Distribution of dataset by the number of images-per-instance**		
3	1001 (23.11%)	3003 (12.26%)
4	853 (19.69%)	3412 (13.93%)
5	387 (8.93%)	1935 (7.90%)
6	1027 (23.71%)	6162 (25.15%)
7	507 (11.70%)	3549 (14.48%)
8	201 (4.64%)	16.08 (6.56%)
9	86 (1.99%)	774 (3.16%)
10+	270 (6.23%)	4059 (16.57%)
Total	4332	24,502

Subcategory	Instances	Images
**(b) Distribution of images and instances by the four subcategories**		
Oriental	2993 (69.09%)	17,471 (71.30%)
Egyptian	820 (18.93%)	4646 (18.96%)
Fulling Mill	309 (7.13%)	1455 (5.94%)
Castle	210 (4.85%)	930 (3.80%)
Total	4332	24,502

### Models

**Figure 3:**
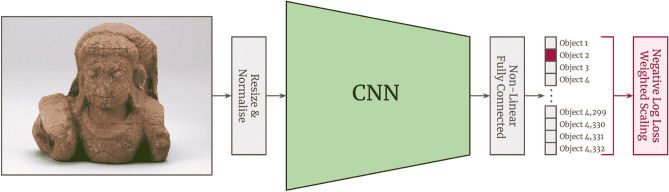
Our end-to-end instance classification training strategy. As the CNN ‘backbone’ is the most substantial processing step, we follow standard practice for image classification tasks and finetune an already-pretrained CNN. This example shows the input image being classified as ‘Object 2’.

We follow modern machine learning practice and take a large CNN that has already been trained on more general image tasks as a starting point, and then further train it on our specific dataset i.e. we ‘finetune’ a ‘pretrained’ state-of-the-art CNN. Our instance classification model (Fig. 3).

takes an image of an object instance as input and outputs a prediction for which object that image belongs to. More formally, our experimental setup is comprised of 4 sequential steps: (1) we use an RGB image of an object (resized to match the resolution used to train the CNN, see Supplementary Table 1) as input to the CNN, which outputs a ‘feature vector’ that represents the information in the image; (2) the feature vector representation is then further processed into a vote for the instance in the image by sequentially passing through further neural network layers: a batch normalisation layer^22^, a fully-connected linear layer, a ReLU non-linearity, a dropout layer^23^ of 0.5, and a final fully-connected linear layer that ultimately outputs a vector with dimension *O* where *O* is the number of object instances (i.e. classes); (3) the *O*-dimensional vector is passed through a log-softmax function, outputting an *O*-dimensional vector that represents a vote for each of the potential *O* objects the input image belongs to; (4) finally, we update the weights in the network by calculating and backpropagating the negative log likelihood ‘loss’ function of the *O*-dimensional vote-vector given the ‘ground truth’ correct answer. Given the class imbalance in our dataset (i.e. variations in the number of images per instance), we weight the loss function for each class (object) by the inverse of its relative occurrence such that objects with fewer images are weighted higher. We use the state-of-the-art EfficientNet^5^ and ResNet Rescaled^6^ as backbones for our instances classifiers, and further ablate with Inception-v3^7^/v4^8^. See Section B of the supplementary materials for more details of our training setup.

## Results

The following subsections provide an extensive ablation study by varying both the makeup of the dataset, and the CNN backbone used in our model.

### Images-per-instance

**Figure 4:**
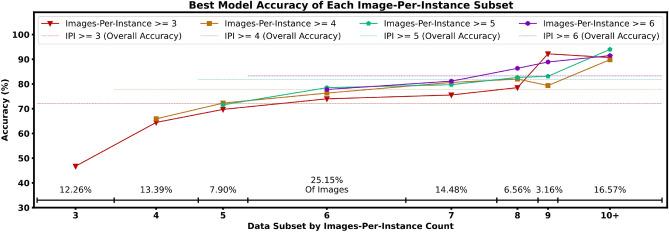
The accuracy of images of each subset by image-per-instance count for our best model (from Fig. 5) under each dataset makeup. For example, the triangular red plots indicate the object prediction accuracy of our highest performing model trained on the dataset with a minimum image-per-instance cutoff of three, further split by subsets of objects with *exactly* 3/4/5... images-per-instance. The dashed red line represents the *overall* accuracy, i.e. a weighted average of accuracies of each subset.

It is important to distinguish the model’s instance classification accuracy for objects with *many* images from those with *fewer* images, because as the number of different images-per-instance increases, so too does the potential visual information for the model to learn to recognise also increase. Given the advantages that more images per instance can afford, in addition to experimenting with the full dataset of 24,502 images (obtained by keeping only instances with 3 images or above as described in “Dataset” section), we also experiment with further restricting the minimum number of images per instance in the dataset to 4, 5, and 6. As shown by the general increase in accuracy at each image-per-instance subset in Fig. 4, we find that instances with a higher number of images are more accurately classified, as intuitively expected. We find that excluding instances with fewer images per instance does *not* substantially improve the accuracy of the higher image count subsets, demonstrated by the relatively close scores of each subset (the most inclusive training setup even scoring the highest on the subset of 9 images-per-instance). This indicates there is little risk to the overall performance of an object detector in including objects of lower image counts, as the lower overall accuracy stems from the naturally poorer classification performance of less represented objects, and *not* a degradation in performance on higher-count subsets. Though it is generally the case that performance increases as the dataset becomes smaller (and thus more easily solved), the similar performance between the four training setups in Fig. 4 imply that our strictest dataset limitation of 6 images per instance has *not* sufficiently reduced dataset size for this problem to manifest. Furthemore, we verify that the high accuracies of our models does not come from a small number of easy or ‘solved’ instances disproportionately carrying the overall accuracy, as demonstrated by the relatively small difference in accuracy between the subsets of image-per-instance counts 8, 9, and 10+.

**Table 3:** Results for the Oriental Museum Dataset test split.

Model	Precision (%)	Recall (%)	F1 (%)	Accuracy (%)	Top-3 Acc (%)	Top-5 Acc (%)	Top-10 Acc (%)
**Images per instance ≥ 3**							
EfficientNet-b0^5^	66.86	89.30	66.21	67.39	78.41	82.04	86.00
EfficientNet-b1^5^	68.04	89.52	67.51	67.71	78.45	82.41	86.37
EfficientNet-b2^5^	67.88	89.77	67.53	67.80	78.82	82.41	86.41
EfficientNet-b3^5^	71.27	90.99	70.83	71.27	81.59	84.90	88.04
EfficientNet-b4^5^	71.31	91.47	70.74	71.18	80.86	84.54	87.88
EfficientNet-b5^5^	71.18	91.26	70.46	71.59	80.57	84.45	88.00
**EfficientNet-b6**^5^	72.37	91.02	71.46	**72.12**	**82.65**	**85.67**	88.90
EfficientNet-b7^5^	71.14	91.30	70.57	71.59	82.04	84.94	88.53
ResNet-RS^6^	51.22	79.77	49.97	50.41	63.96	68.41	74.65
Inception-v3^7^	59.84	85.20	59.32	59.47	72.49	76.90	82.16
Inception-v4^8^	59.76	84.70	59.09	59.76	72.16	76.29	80.73
***Ensemble (All)***	66.86	89.30	66.21	70.73	80.98	83.63	87.84
***Ensemble (ENet-b[3-7])***	71.27	90.99	70.83	**73.22**	81.96	84.73	88.73
**Images per instance ≥ 4 **							
EfficientNet-b0^5^	74.33	90.11	73.28	74.28	84.37	87.30	90.76
EfficientNet-b1^5^	74.33	90.90	73.52	74.60	85.02	87.95	91.30
EfficientNet-b2^5^	75.63	90.67	74.69	75.44	85.12	88.00	91.16
**EfficientNet-b3**^5^	77.21	92.59	76.68	**77.86**	87.12	89.81	92.23
EfficientNet-b4^5^	76.93	91.99	76.45	76.98	87.12	89.58	92.28
EfficientNet-b5^5^	77.16	91.75	76.28	77.72	86.23	89.21	92.14
EfficientNet-b6^5^	77.49	92.19	76.91	77.58	**87.63**	**90.23**	**92.88**
EfficientNet-b7^5^	75.95	91.95	75.13	77.12	86.23	89.49	92.56
ResNet-RS^6^	59.63	83.31	58.86	58.60	71.44	76.09	80.93
Inception-v3^7^	63.95	86.06	62.89	64.09	76.37	80.00	85.21
Inception-v4^8^	64.00	85.82	63.16	64.23	76.47	79.72	85.12
***Ensemble (All)***	74.33	90.11	73.28	76.79	85.40	88.05	90.98
***Ensemble (ENet-b[3-7])***	77.21	92.59	76.68	**78.47**	86.84	89.81	92.84
**Images per instance ≥ 5**							
EfficientNet-b0^5^	78.46	93.06	78.25	78.52	87.24	89.45	92.77
EfficientNet-b1^5^	78.74	92.56	78.67	78.80	87.58	90.23	92.49
EfficientNet-b2^5^	78.69	93.17	78.09	79.29	88.07	90.72	93.59
EfficientNet-b3^5^	81.39	93.77	81.22	81.06	89.40	91.94	93.87
**EfficientNet-b4**^5^	81.94	94.64	81.98	**81.83**	**90.50**	92.21	94.70
**EfficientNet-b5**^5^	82.05	94.22	81.90	**81.83**	89.62	91.99	94.20
EfficientNet-b6^5^	81.34	93.54	81.21	81.34	89.67	91.99	94.42
EfficientNet-b7^5^	81.17	94.39	80.76	81.17	90.45	**92.71**	**94.92**
ResNet-RS^6^	62.73	86.04	62.44	61.07	72.45	76.92	82.05
Inception-v3^7^	67.31	87.61	66.70	64.94	77.75	82.61	86.31
Inception-v4^8^	66.37	87.00	66.14	66.65	77.53	81.28	85.92
***Ensemble (All)***	78.46	93.06	78.25	82.55	89.29	91.39	93.87
***Ensemble (ENet-b[3-7])***	81.39	93.77	81.22	**83.77**	90.28	92.32	94.86
**Images per instance ≥ 6**							
EfficientNet-b0^5^	80.19	92.91	79.73	80.12	88.24	90.90	93.68
EfficientNet-b1^5^	79.44	92.15	79.20	79.44	87.18	89.97	93.31
EfficientNet-b2^5^	80.62	92.47	80.13	80.43	89.29	91.08	93.75
EfficientNet-b3^5^	82.66	93.50	82.34	82.11	90.46	92.69	**94.80**
EfficientNet-b4^5^	81.55	93.49	81.47	81.55	90.03	92.26	94.67
EfficientNet-b5^5^	82.17	92.77	82.04	82.54	89.72	91.83	95.36
**EfficientNet-b6**^5^	83.41	93.36	83.01	**83.28**	**90.53**	**92.82**	**94.80**
EfficientNet-b7^5^	80.50	93.03	80.31	80.06	89.47	91.89	94.30
ResNet-RS^6^	65.20	85.48	64.40	63.53	77.89	81.55	86.01
Inception-v3^7^	67.55	87.26	67.02	67.55	80.43	84.09	88.48
Inception-v4^8^	67.80	86.19	67.27	67.80	79.26	82.48	86.44
***Ensemble (All)***	80.19	92.91	79.73	82.79	90.03	92.45	94.18
***Ensemble (ENet-b[3-7])***	82.66	93.50	82.34	**84.02**	90.84	93.37	95.05

*‘Ensemble’* refers to the score dervied using the *average* of class votes for *all* of the models. *‘Ensemble b[3-7]’* considers the strongest models only i.e. EfficientNet-b3,4,5,6,7.

Significant values are in bold.

### CNN model

**Figure 5:**
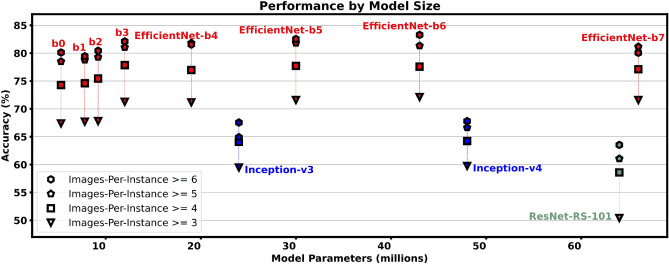
Performance of models with respect to it’s size i.e. the number of parameters. The four dataset image-per-instance scenarios here are the same four scenarios as in Fig. 4.

As the CNN backbone is the most important part of the model design, we experiment with state-of-the-art EfficientNet^5^ and ResNet Rescaled^6^ models with further ablation on Inception-v3^7^/v4^8^ models. We find that all EfficientNet models perform significantly better than the other models on each training setup (Fig. 5), and that the larger EfficientNet models consistently achieve a higher accuracy than the smaller ones. Though a higher image-per-instance threshold yields gives higher performance as previously discussed, we also see that the model size does not significantly change the performance on *different image-per-instance thresholds*. This is depicted by the consistent spacing between each polygon in Fig. 5 across each different type of model. Instead, the change in accuracy at image-per-instance thresholds is noticeably different for the three different model *architectures*, i.e. the increase in performance from images-per-instace ≥ 3 and ≥ 6 is $\sim$ 19%, $\sim$ 11%, and $\sim$ 8% for ResNet-RS, EfficientNet, and Inception architectures respectively.

### High performance trade-offs

The strongest performing *single* model is EfficientNet-b4/3/4/6 for image-per-instance dataset thresholds ≥ 3/4/5/6 respectively. However, to maintain an adequately large batch size for stable training, the larger EfficientNet variations require significantly larger computational resources during *training* (full details in Supplementary Table 1). Furthermore, inline with standard practice for CNN-based image classification models^24^, we find that a collaborative ensemble of models pushes accuracy even higher. Table 3 shows that an ensemble of the 5 best EfficientNet models gives a ~ 1–2% increase in top-1 accuracy, which can be exploited provided one is willing to purchase it with increases in computational resources (~ 2.5 GB of VRAM for *inference* of a single image). Even in scenarios where the predicted object is incorrect, we find that the correct answer is often still in the next few guesses, i.e. the top-3, top-5, and top-10 accuracies of our models are actually significantly higher than our regular accuracy scores. We see in Table 3 that (for the best performing *single* model) the more-relaxed ‘image-per-instance ≥ 3/4′ dataset scenarios yield ~ 10%, ~ 13%, and ~ 17% improvements for top-3, top-5 and top-10 accuracies respectively. The top-3, top-5, and top-10 accuracies show less relative improvement for the less relaxed image-per-instance ≥  5/6 scenarios (+ ~ 8%, + ~ 10%, + ~ 12%) as the baseline top-1 accuracies are already higher than image-per-instance ≥ 3/4.

### Subcategories of objects

**Figure 6:**
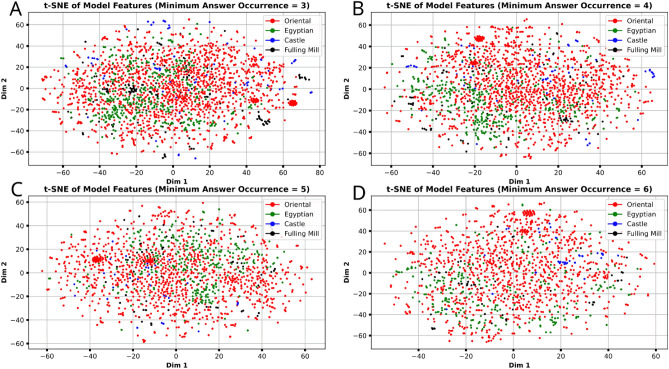
t-SNE Dimension Reduction^25^ on the features generated from each image of the dataset, extracted from the penultimate layer of the CNNs used in our experiments.

Though the central question of this paper is about instances, we explore how the instance classification accuracy differs for instances in each subcategory of the dataset. We see in Table 4 that the Oriental and Egyptian subcategories score consistently above the overall average. Castle objects score significantly below average (− 8% to − 32%), and Fulling Mill objects score between (− 8% to + 0.33%). The biggest variations in subcategory accuracy occur in the more relaxed image-per-instance ≥ 3/4 scenarios (+ 6% to − 29.%), whereas the more restrictive and generally higher-performing image-per-instance ≥ 6 scenario has much less variation overall (+ 1% to − 8%). We note that the smaller subcategories experience the most substantial drop in accuracy, and that this further coincides with the average image-per-instance for each subsection (calculated from Table 2b): Oriental $\approx$  5.84, Egyptian $\approx$  5.67, Fulling Mill $\approx$  4.71, Castle $\approx$ 4.43. However, we cannot conclude that the larger size of a subcategory is the cause of increasing performance as the Egyptian subcategory (~ 18.93% of instances) scores higher than the much larger Oriental subset (~ 69.09% of instances). Conversely, we also cannot conclude that the relatively small size of the Fulling Mill ($\sim$ 5.94%) and Castle ($\sim$ 3.80%) subcategories cause their relative reduction in performance compared with overall accuracy, because the accuracy of these two smaller subcategories approaches the overall accuracy in the higher image-per-instance dataset scenarios. We hypothesise that instances of these subcategories are instead not as easily represented with less images-per-instance. In order to gauge the differences in image information between the subsections, we apply t-SNE dimension reduction^25^ on feature vectors extracted from the penultimate layer of the CNN in our best models for each image in test set. This generates a 2D point for each image which we can plot to observe any clusters the t-SNE reduction may have generated. We see from Fig. 6 that the plot the 2-dimensional t-SNE reduction generates does *not* strongly cluster the images by *subsets*, as the four colours (representing each different subcategory) are relatively evenly distributed. However, the points instead appear cluster into a large number of very small neighbourhoods *irrespective* of their subcategory. We find this unsurprising, as the CNN has been trained to distinguish images by *instance* instead of their subcategory. This is evidence that our model is *not* relying on features unique to each subcategory *e.g. Oriental*, and is instead primarily using the distinctive features of each *object instance* as intended. See Supplementary Figs. 2 and 3 in Section D of the supplementary materials for PCA and UMAP dimensionality reduction respectively.

**Table 4:** Accuracy of the highest overall performing model for instance classifications from *each of the subsets* comprising the whole dataset.

Subset	Precision (%)	Recall (%)	F1 (%)	Accuracy (%)	Top-3 Acc (%)	Top-5 Acc (%)	Top-10 Acc (%)
**Images per instance ≥ 3 (best model = ENet-b6)**							
Overall	72.37	91.02	71.46	72.12	82.65	85.67	88.90
Oriental	72.63	91.40	71.84	**+ 0.62** 72.74	83.19	86.25	89.50
Egyptian	79.07	94.69	79.20	**+ 6.35** 78.47	87.32	89.74	92.15
Fulling Mill	71.24	94.90	71.79	***− 2.19*** 69.93	84.31	86.93	90.20
Castle	47.06	83.33	48.27	***− 28.59*** 43.53	61.18	68.24	76.47
**Images per instance ≥ 4 (best model = ENet-b3)**							
Overall	77.21	92.59	76.68	77.86	87.12	89.81	92.23
Oriental	78.05	93.23	77.63	**+ 0.77** 78.63	87.62	90.42	92.38
Egyptian	80.68	96.33	81.15	**+ 2.82** 80.68	90.23	91.82	93.41
Fulling Mill	75.47	93.50	75.92	***− 0.50*** 77.36	85.85	85.85	91.51
Castle	40.91	71.95	41.67	***− 32.41*** 45.45	60.61	72.73	86.36
**Images per instance ≥ 5 (best model = ENet-b4)**							
Overall	81.94	94.64	81.98	81.83	90.50	92.21	94.70
Oriental	82.16	94.78	82.08	**+ 0.18** 82.01	90.40	92.28	94.90
Egyptian	85.03	97.10	85.92	**+ 3.20** 85.03	93.22	94.07	95.20
Fulling Mill	74.12	99.32	77.88	***− 7.71*** 74.12	87.06	89.41	92.94
Castle	63.16	92.86	64.64	***− 18.67*** 63.16	76.32	78.95	86.84
**Images per instance ≥ 6 (best model = ENet-b6)**							
Overall	83.41	93.36	83.01	83.28	90.53	92.82	94.80
Oriental	83.54	94.39	83.48	**+ 0.01** 83.29	90.50	92.47	94.68
Egyptian	83.96	95.74	84.53	**+ 1.02** 84.30	91.47	94.20	95.56
Fulling Mill	83.61	94.35	83.61	**+ 0.33** 83.61	90.16	95.08	96.72
Castle	75.00	86.84	77.74	***− 8.28*** 75.00	85.00	90.00	90.00

Significant values are in bold. We provide the difference in accuracies between each individual class and the overall accuracy.

### Visualising predictions with saliency maps

**Figure 7:**
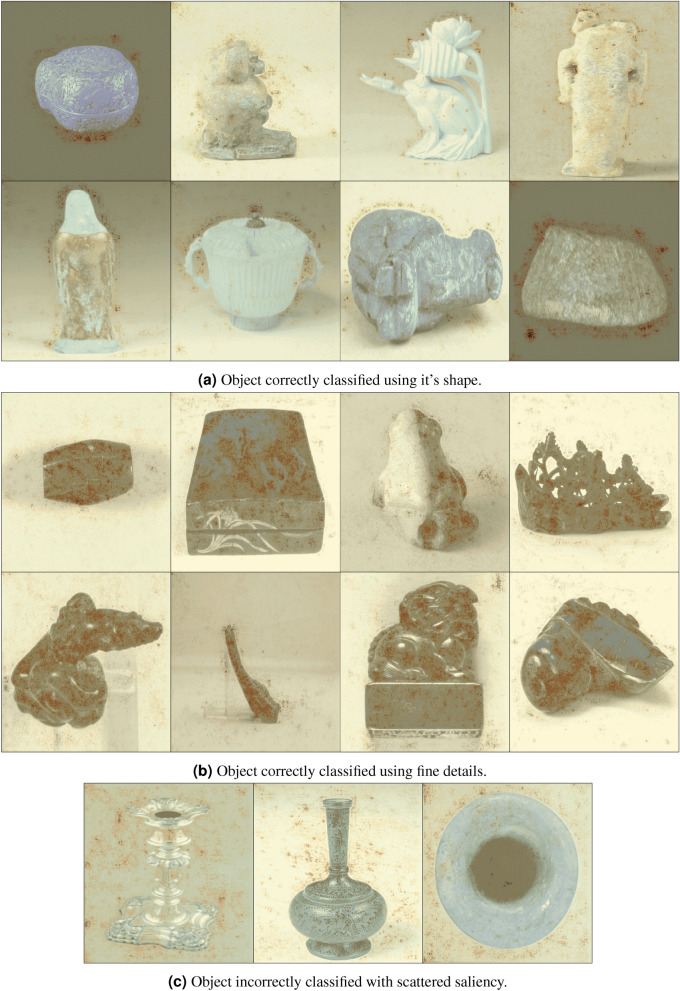
Saliency maps^26^ generated from our best model on images-per-instance ≥ 6 (EfficientNet-b6) to visualise which regions in the image are most influential in choosing the instance. The saliency map is overlaid on the original image for clarity. The darker red regions indicate a higher intensity score.

We use saliency maps^26^ to see which regions of the input image were most influential in the decision the network makes, and thus allowing us to estimate how our neural network makes prediction for which instance it believes the image belongs to. Given an image and the class a model has predicted for it, we can track the origin of the signals propagating through the network that led to the given classification, i.e. we can highlight the image regions that most influenced the model’s instance classification choice. Figure 7 shows saliency maps generated by our best single model (EfficientNet-b6 on images-per-instance ≥ 6) overlaid on the original image for clarity. A higher intensity (darker red) saliency indicates that the pixels were highly influential in the model’s decision. Our models do not demonstrate an over-reliance on any one feature in its predictions: Fig. 7a shows examples where the boundary of the object, i.e. its shape, led to correct classification. Figure 7b instead shows examples where the finer details on the surface of the objects is most salient to correct classification. We did not find any one feature in saliency that correlates with *incorrect* classifications. However, it is often the case that *incorrectly* classified objects exhibit a more scattered saliency as in Fig. 7c. Such incorrect saliency maps still appear to somewhat attend to the shape/details of the objects, though to a much lesser degree than the correctly classified counterparts in Fig. 7a,b. For example, objects in Fig. 7c demonstrate regions of saliency spread thinly across the background. Yet *some* of the saliency is still overlaid around the shape of the objects (middle and right objects), or the details (seen on the base of the leftmost object in Fig. 7c). This behaviour is typical of a less confident prediction, where the model is still aware of the features in the object, but is unable to exploit them confidently in classification.

## Discussion

Our experimental results represent a strong proof-of-concept for a framework to tackle the illicit movement of cultural heritage through the use of highly accurate and flexible instance classification. Though our findings indicate that greater performance for instance classification can be unlocked from larger CNNs (“CNN model” section) and more data (“Images-per-instance” section), our strong results with smaller CNNs—which have a relatively small computational footprint—indicate this framework is ideal for deployment applications on mobile devices. Moreover, a large increase in the number of instances and images at training time does *not* significantly increase the computational footprint of the framework at inference time (deployment), allowing our approach to scale with the amount of data available. The top-1 accuracy of our framework is designed to be an automatic detection system without the need for manual oversight. However, if so desired, our framework allows the flexibility to purchase even higher instance detection (top-k) accuracy by presenting the first *k* objects predicted for manual consideration. The top-k accuracies of our ensemble of the strongest models are either equal-to, or slightly stronger than the top-k accuracies of our best single model. The strongest *single* model remains competitive with such an ensemble, however this performance boost is available in any scenario where computational resources are not a bottleneck. Figure 4 shows that the biggest increase in performance for classifying an instances comes in the jump between three and four images-per-instance, with diminishing returns after around seven, and eight. These results offer a minimum ‘ideal number’ of images-per-instance to aim for, and the respective accuracy that can be expected if resources are limited. Though we find some variation in the instance classification accuracy between objects of the four different subcategories in our images-per-instance ≥ 3/4 dataset scenarios, this difference rapidly decreases as the minimum number of image-per-instance increases. This implies that though different artefact types can perform worse with less images-per-instance, increasing the number of images-per-instance is a good countermeasure. The uniform backgrounds in the images we use denies our model the opportunity to exploit background-bias shortcuts and ensures our instance classification scores are reliable, but it also in turn limits this dataset’s applicability to scenarios with noisier backgrounds. We explore this point further in “Limitations” section. As outlined in “Subcategories of objects” section, we have evidence that predictions are truly considering the individual instances and not the overall subcategories.

## Limitations

Though a dataset of 24,502 images is reasonably large for a domain-specific computer vision task, it is still orders of magnitude smaller than other computer vision datasets (*e.g.* ImageNet^27^) that contend with the largest of modern architectures. The large EfficientNet models can classify images during *inference* (deployment) relatively rapidly and with relatively low memory costs. However, during the *training* process where the neural network must update all of its weights, the largest EfficientNet models demand much more memory in order to be trained with an adequately large batch size i.e. NVIDIA A100. The smaller EfficientNet models *e.g.* can however be trained *and* inferred with much more reasonable computational resources with very little performance degradation. We have specifically curated this dataset from images of objects in a variety of different poses and angles. However, the backgrounds of the objects are uniform, reflecting the controlled environment that these images were collected in. Though this *prevents* our model from learning to exploit background bias shortcuts, it does however mean that this dataset alone would not generalise well to a real world setting. The uniform background settings and varying poses of this dataset allows us to accurately demonstrate our model’s instance classification capability, but it also follows that this dataset would need to be supplemented with other ‘noisier’ images that appropriately control for the background biases that will be encountered in each individual deployment scenario.

## Conclusion

We introduce a framework for accurately detecting the exact object instance of an image from amongst thousands of others. Our approach represents a strong proof-of-concept for an application to detect illicit movement of cultural heritage. We achieve 73% instance classification accuracy on a diverse dataset of images of artefacts with 24,502 images and 4332 unique object instances, increasing up to 83% with higher images-per-instance counts. Our approach offers two potential trade-offs: 84% accuracy for a higher computational footprint through an ensemble of the strongest models; or 95%+ accuracy by introducing a small number of objects for manual review. Our results demonstrate the potential in the overlap between cultural heritage and machine learning, and suggest even greater performance that is immediately available given an even larger and more diverse dataset.

## Supplementary Information

Supplementary Information.

## Data Availability

The data used in our study is from the digital collection of the Durham Oriental Museum, and access can be requested here: https://www.dur.ac.uk/oriental.museum/contact/. Our implementation is hosted in our GitHub repository https://github.com/Durham-University-VIVID-Noura-s-Lab/artefact_instance_cls.
