# Supplementary material for: A deep learning approach to fight illicit trafficking of antiquities using artefact instance classification
Source: Sci Rep. 2022 Aug 5;12:13468. doi: 10.1038/s41598-022-15965-2 (PMC9356139; doi:10.1038/s41598-022-15965-2)
Supplement: Supplementary file 1 — Supplementary Information. [file 41598_2022_15965_MOESM1_ESM.pdf]

A Image Resolutions

Supplementary Figure 1 shows the resolutions of images in that dataset, and the input resolutions that these images are resized to in training each different model.

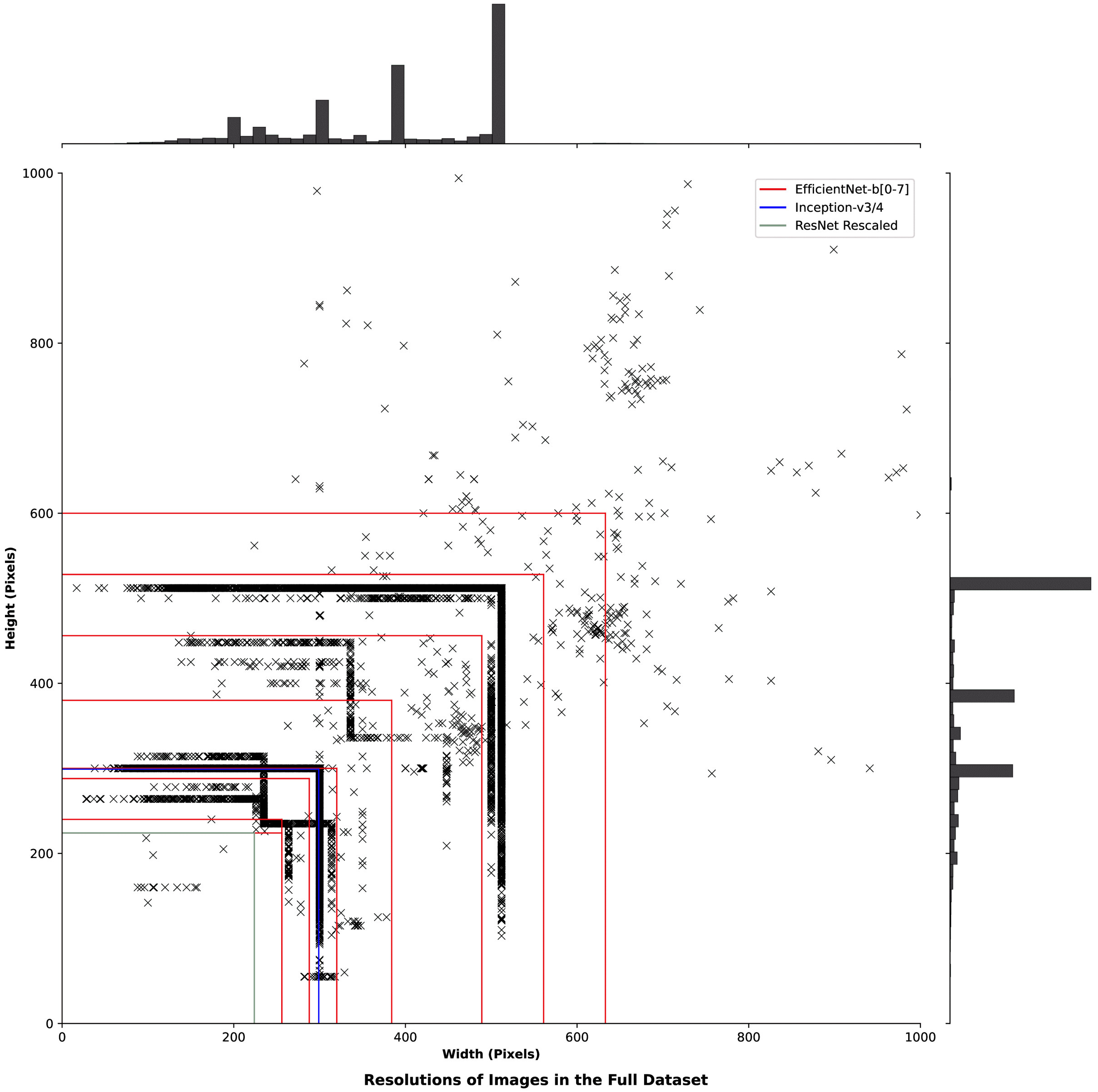

Supplementary Figure 1. The distribution of image resolutions in the dataset. The coloured boxes indicate the image resolution that each CNN is trained with (*i.e.* images are resized to the resolutions each CNN is originally pretrained with). The resolution used for EfficientNet models monotonically increases with the model size (and name; b0, b1, ..., b7). See Supplementary Table 1 for the exact values of the plotted resolutions. Note that this plot represents ∼97% of all images, the remaining ∼3% images have either height or width greater than 1,000 pixels.

B Further Training Details

We use the Adam optimiser[1] with a learning rate = $10^{-4.8}$, loss weight scaling. Models are trained for 100 epochs. See Section C for details on batch sizes and image resolutions for each model. Models with EfficientNet-B3, B4, B5, B6, and B7 are trained on NVIDIA A100 GPUs. All other model types are trained on either an NVIDIA 2080 Ti or NVIDIA Titan Xp GPU. The intermediate fully-connected layer used after batch normalisation is of dimension 2,048. Our models are finetuned using

pretrained CNNs available from Torchvision[2]. Unless otherwise specified, our dataset is randomly but deterministically shuffled using NumPy's implementation of the PCG64 generator[3] with seed = 2,667.

C Model Resolutions and Batch Sizes

We find that the models used in our experiment yield the highest accuracy when the resolution of their input images matches the resolution they were pretrained with. In general, the larger models also use larger image resolutions, which limits the maximum batch size we can use in training. See Supplementary Table 1 for full details.

Model	Image Resolution	Batch Size	GPU (VRAM)
EfficientNet-B0	(256, 224)	32	2080Ti/TitanXP (11GB/12GB)
EfficientNet-B1	(256, 240)	32	2080Ti/TitanXP (11GB/12GB)
EfficientNet-B2	(288, 288)	32	2080Ti/TitanXP (11GB/12GB)
EfficientNet-B3	(320, 300)	32	2080Ti/TitanXP (11GB/12GB)
EfficientNet-B4	(384, 380)	32	A100 (80GB)
EfficientNet-B5	(489, 456)	32	A100 (80GB)
EfficientNet-B6	(561, 528)	32	A100 (80GB)
EfficientNet-B7	(633, 600)	16	A100 (80GB)
ResNet-RS	(224, 224)	32	2080Ti/TitanXP (11GB/12GB)
Inception-v3	(299, 299)	32	2080Ti/TitanXP (11GB/12GB)
Inception-v4	(299, 299)	32	2080Ti/TitanXP (11GB/12GB)

Supplementary Table 1. The image resolution and batch size used to train each model.

D Image Feature Clustering

Similar to Figure 6 from the main paper, Supplementary Figures 2 and 3 show the 2-dimensional reduction of feature vectors extracted from the penultimate layer of the CNN in our best models for each image in test set for PCA and UMAP dimensionality reduction respectively.

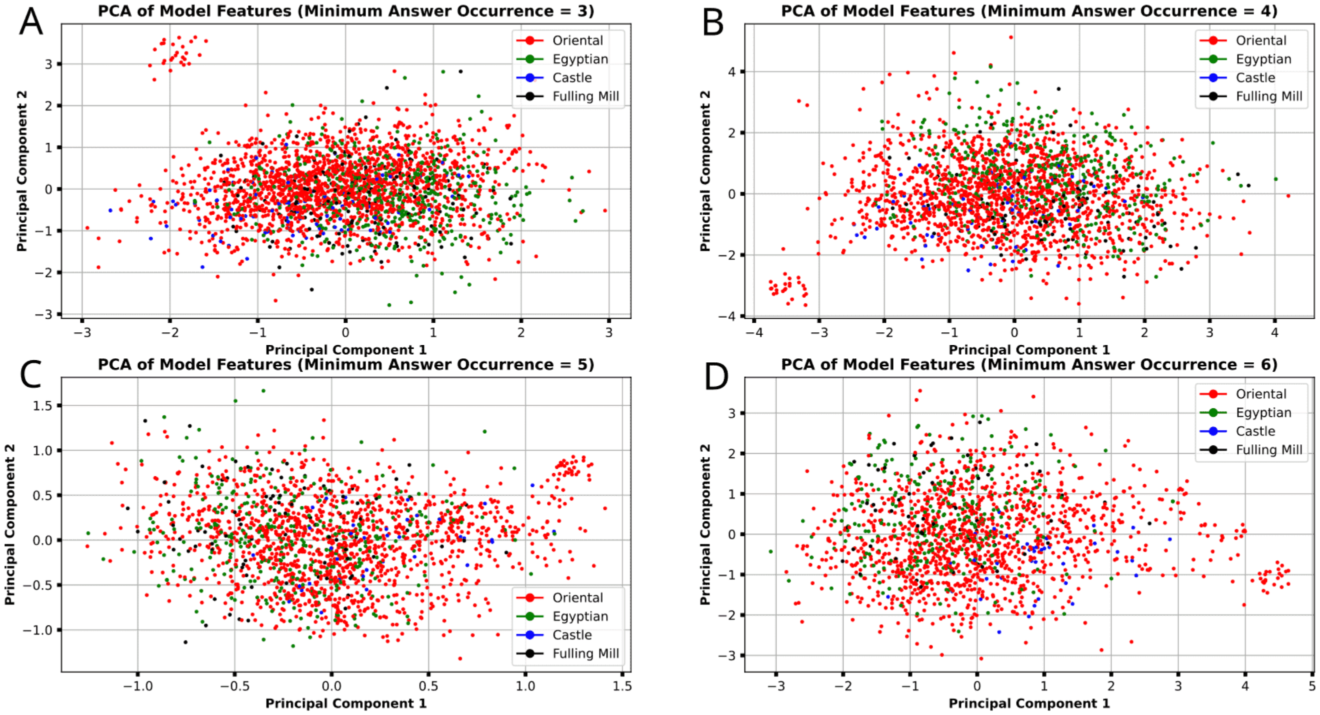

Supplementary Figure 2. Principal Component Analysis Dimension Reduction[4] on the features generated from each image of the dataset, extracted from the penultimate layer of the CNNs used in our experiments.

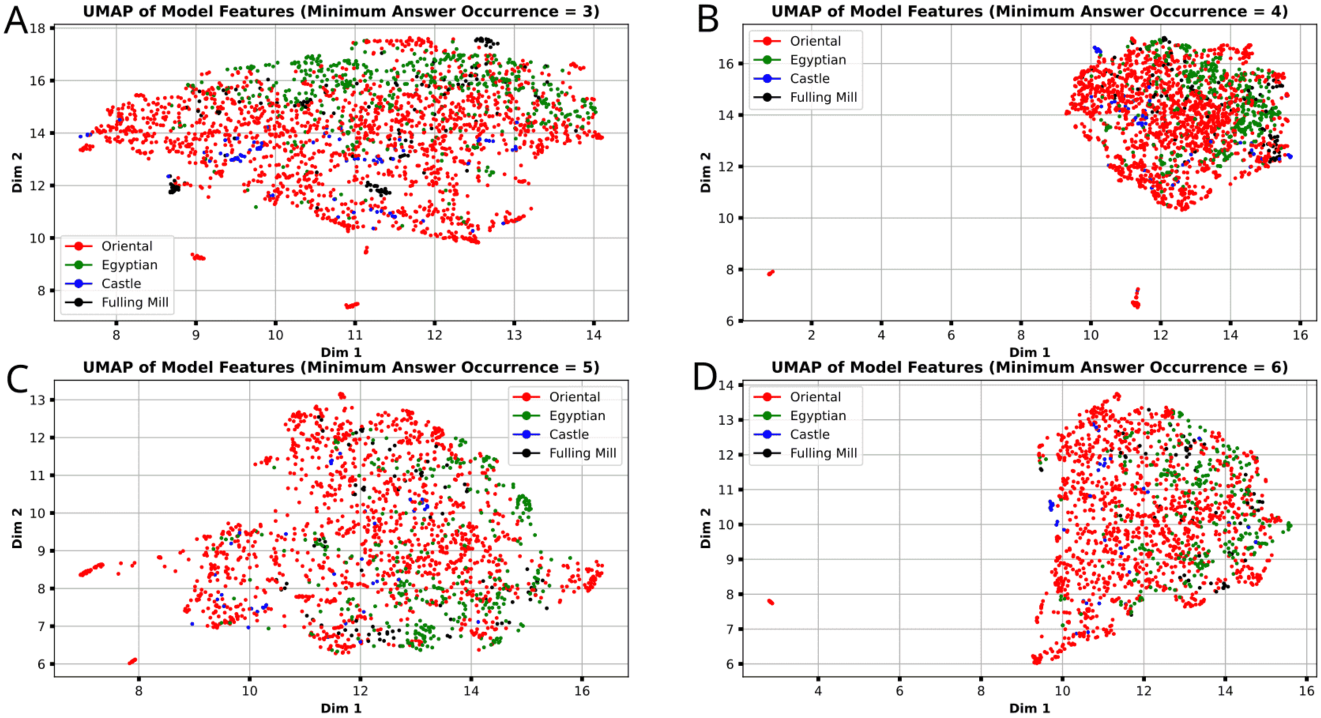

Supplementary Figure 3. UMAP Dimension Reduction[5] on the features generated from each image of the dataset, extracted from the penultimate layer of the CNNs used in our experiments.

E Run Time

Supplementary Table 2 shows the run time for each of our experiments in Table 3 from the main paper.

	Model	Run Time (Days-Hours:Minutes:Seconds)
Images Per Instance >= 3	EfficientNet-b0[6]	2:02:25
	EfficientNet-b1[6]	2:50:12
	EfficientNet-b2[6]	3:46:07
	EfficientNet-b3[6]	3:53:31
	EfficientNet-b4[6]	5:50:13
	EfficientNet-b5[6]	11:37:01
	**EfficientNet-b6[6]**	**16:51:05**
	EfficientNet-b7[6]	1-04:35:37
	ResNet-RS[7]	7:21:38
	Inception-v3[8]	5:23:59
	Inception-v4[9]	10:03:23
	Ensemble (All)	9:38
	Ensemble (ENet-b[3-7])	**5:37**
Images Per Instance >= 4	EfficientNet-b0[6]	1:47:11
	EfficientNet-b1[6]	2:29:23
	EfficientNet-b2[6]	3:14:28
	**EfficientNet-b3[6]**	**3:26:01**
	EfficientNet-b4[6]	5:05:17
	EfficientNet-b5[6]	9:25:45
	EfficientNet-b6[6]	14:26:53
	EfficientNet-b7[6]	1-01:02:06
	ResNet-RS[7]	6:30:30
	Inception-v3[8]	4:45:11
	Inception-v4[9]	8:49:10
	Ensemble (All)	10:20
	Ensemble (ENet-b[3-7])	**4:54**
Images Per Instance >= 5	EfficientNet-b0[6]	1:29:12
	EfficientNet-b1[6]	2:05:10
	EfficientNet-b2[6]	2:45:44
	EfficientNet-b3[6]	3:47:34
	**EfficientNet-b4[6]**	**4:13:42**
	EfficientNet-b5[6]	7:43:49
	EfficientNet-b6[6]	†20:03:12
	EfficientNet-b7[6]	†1-02:08:16
	ResNet-RS[7]	5:24:35
	Inception-v3[8]	4:04:01
	Inception-v4[9]	7:25:38
	Ensemble (All)	9:33
	Ensemble (ENet-b[3-7])	**6:06**
Images Per Instance >= 6	EfficientNet-b0[6]	1:27:25
	EfficientNet-b1[6]	1:57:04
	EfficientNet-b2[6]	2:31:19
	EfficientNet-b3[6]	3:17:01
	EfficientNet-b4[6]	4:32:46
	EfficientNet-b5[6]	7:26:01
	**EfficientNet-b6[6]**	**11:43:02**
	EfficientNet-b7[6]	19:18:08
	ResNet-RS[7]	4:53:48
	Inception-v3[8]	3:38:27
	Inception-v4[9]	6:45:22
	Ensemble (All)	7:09
	Ensemble (ENet-b[3-7])	**5:47**

Supplementary Table 2. Run times for the Oriental Museum Dataset experiments in Table 3 from the main paper. ***'Ensemble'*** refers to the score dervied using the *average* of class votes for *all* of the models. ***'Ensemble b[3-7]'*** considers the strongest models only *i.e.* EfficientNet-b3,4,5,6,7. Note the ***'ensemble'*** methods in this table use the other models in this table (already trained), and therefore only run evaluation on the test set. † indicates a run slower than otherwise expected due to coinciding network overhead during experiments.

References

1. Kingma, D. & Ba, J. Adam: A method for stochastic optimization. *Int. Conf. on Learn. Represent.* (2014).
2. PyTorch torchvision models. https://pytorch.org/vision/stable/models.html. Accessed: 11-02-2022.
3. NumPy permuted congruential generator (64-bit, pcg64). https://numpy.org/doc/stable/reference/random/bit_generators/pcg64.html. Accessed: 11-02-2022.
4. Pearson, K. Liii. on lines and planes of closest fit to systems of points in space. *The London, Edinburgh, Dublin philosophical magazine journal science* **2**, 559–572 (1901).
5. McInnes, L., Healy, J. & Melville, J. Umap: Uniform manifold approximation and projection for dimension reduction. *arXiv preprint arXiv:1802.03426* (2018).
6. Tan, M. & Le, Q. V. Efficientnet: Rethinking model scaling for convolutional neural networks. *ArXiv* **abs/1905.11946** (2019).
7. Bello, I. *et al.* Revisiting resnets: Improved training and scaling strategies. In Beygelzimer, A., Dauphin, Y., Liang, P. & Vaughan, J. W. (eds.) *Advances in Neural Information Processing Systems* (2021).
8. Szegedy, C., Vanhoucke, V., Ioffe, S., Shlens, J. & Wojna, Z. Rethinking the inception architecture for computer vision. In *2016 IEEE Conference on Computer Vision and Pattern Recognition (CVPR)*, 2818–2826, DOI: 10.1109/CVPR.2016.308 (2016).
9. Szegedy, C., Ioffe, S., Vanhoucke, V. & Alemi, A. Inception-v4, inception-resnet and the impact of residual connections on learning. *AAAI Conf. on Artif. Intell.* (2016).
